# Efficient Fabrication of Polycaprolactone Scaffolds for Printing Hybrid Tissue-Engineered Constructs

**DOI:** 10.3390/ma12040613

**Published:** 2019-02-18

**Authors:** Enrique Sodupe Ortega, Andres Sanz-Garcia, Alpha Pernia-Espinoza, Carmen Escobedo-Lucea

**Affiliations:** 1 EDMANS Group, Department of Mechanical Engineering, University of La Rioja, San José de Calasanz 31, Edificio Departamental, 26004 Logroño, Spain; enrique.sodupeo@unirioja.es (E.S.O.); alpha.pernia@unirioja.es (A.P.-E.); 2 Division of Pharmaceutical Biosciences, University of Helsinki, Viikinkaari 5 E (P.O. Box 56), 00014 Helsinki, Finland; 3 Department of Mechanical Engineering, University of Salamanca, ETSII, Avda, Fernando Ballesteros, 2, 37700 Béjar (Salamanca), Spain

**Keywords:** tissue engineering, polycaprolactone, porous scaffolds, hybrid constructs, 3D printing

## Abstract

Hybrid constructs represent substantial progress in tissue engineering (TE) towards producing implants of a clinically relevant size that recapitulate the structure and multicellular complexity of the native tissue. They are created by interlacing printed scaffolds, sacrificial materials, and cell-laden hydrogels. A suitable biomaterial is a polycaprolactone (PCL); however, due to the higher viscosity of this biopolymer, three-dimensional (3D) printing of PCL is slow, so reducing PCL print times remains a challenge. We investigated parameters, such as nozzle shape and size, carriage speed, and print temperature, to find a tradeoff that speeds up the creation of hybrid constructs of controlled porosity. We performed experiments with conical, cylindrical, and cylindrical shortened nozzles and numerical simulations to infer a more comprehensive understanding of PCL flow rate. We found that conical nozzles are advised as they exhibited the highest shear rate, which increased the flow rate. When working at a low carriage speed, conical nozzles of a small diameter tended to form-flatten filaments and became highly inefficient. However, raising the carriage speed revealed shortcomings because passing specific values created filaments with a heterogeneous diameter. Small nozzles produced scaffolds with thin strands but at long building times. Using large nozzles and a high carriage speed is recommended. Overall, we demonstrated that hybrid constructs with a clinically relevant size could be much more feasible to print when reaching a tradeoff between temperature, nozzle diameter, and speed.

## 1. Introduction

In tissue engineering (TE), the use of three-dimensional (3D) printing, also known as additive manufacturing (AM), for the fabrication of scaffolds has steadily increased over the past few years [1,2,3]. Many biocompatible materials have been successfully printed; however, one of the most outstanding synthetic resorbable polymers is polycaprolactone (PCL) due to its mechanical strength, stiffness (enough to influence cell behavior [4]), and tailorable degradation kinetics [5,6]. PCL cannot be directly formulated with cells [7], but many in-vitro studies have shown clear cell spreading, attachment, and extracellular matrix formation over PCL scaffolds [2,8,9]. This biopolymer is also easy to extrude due to its superior viscoelastic and rheological properties over many of its resorbable counterparts [10]. All these features enable the generation of scaffolds with complex geometry and precise control of their internal porosity at a low cost.

Many studies employ electrospinning to generate scaffolds with variable fiber diameters and porosities [11]. Conventional electrospinning is a simple and versatile process to produce 3D fibrous constructs with interconnected pores using a wide range of polymers. These scaffolds have a high surface-area-to-volume ratio that mimics the natural extracellular matrix (ECM) [12]. However, electrospun scaffolds have limitations in creating 3D structures of relevant physiological thickness [13]. An alternative way to facilitate the construction of thicker structures is the use of AM. Such technologies as fused deposition modeling (FDM) [14,15] and microextrusion-based systems [16] are very suitable for extruding PCL to produce 3D scaffolds with controlled porosity through a layer-by-layer approach. FDM is a technology particularly useful for filament-type polymers of very low viscosity at a high temperature, such as polylactic acid (PLA) or acrylonitrile butadiene styrene (ABS). FDM has been used to produce 3D scaffolds of PCL [17]. However, when creating hybrid constructs, microextrusion-based systems are preferred because of the deposition of molten PCL at a lower temperature but a higher pressure than FDM. Two types of extrusion-based systems are currently employed: pneumatic and mechanical. As the viscosity and strength of PCL depend on its molecular weight [18], mechanical-based systems, which generate a higher extrusion pressure, are preferred for printing PCL with high molecular weight [19,20]. On the other hand, PCL with a low or intermediate molecular weight can be extruded with pneumatic systems due to lower pressure requirements [21,22]. Herein, we used a pneumatic printhead, built in-house, for extruding PCL. The printhead is similar to the ones used for cell bioprinting, but with the capacity to work at significantly higher pressures and print temperatures above 80 °C.

Novel AM systems with the capacity to print biomaterials and cell-laden hydrogels in a single session have gained much attention for their potential application in the generation of complex human-scale tissue constructs of any shape, such as calvarial bone, cartilage, and skeletal muscle [23,24,25,26]. These so-called “printed hybrid TE constructs” combine 3D scaffolds of complex architectures, cell-laden bioinks extruded in integrated patterns, and microchannels that allow for the diffusion of nutrients and oxygen across the construct. The hybrid TE constructs aim to assure cell survival and enough structural integrity for surgical implantation. The bioinks support cell survival and proliferation, and the scaffold provides enough structural integrity and mechanical resistance for surgical implantation [27]. Building hybrid constructs requires AM systems with multiple printheads, precise calibration, and accurate print temperature control [28]. The rapid generation of the hybrid constructs is critical for reducing the total print time. Long print times dramatically decrease cell viability [29]. Shorter ones are preferred but without decreasing print accuracy because precise material deposition is also essential for cell viability. Besides, cell viability also decreases when printing the new layers of the scaffold at a high temperature over areas of the printed hybrid construct [23]. Therefore, we must reach a balance between print resolution, temperature, and carriage speed by finding the most appropriate print parameters for each specific application.

In this paper, we studied the possible values of specific print parameters that could help in generating supporting structures of PCL for hybrid constructs while minimizing the print time. The primary candidates, which have a direct influence on the PCL flow rate, were print temperature, nozzle shape and diameter, carriage speed, and inlet pressure. For the first time, we investigated the differences in flow using three types of nozzles with different diameters on the same 3D printing platform: conical, cylindrical, and cylindrical shortened. Experiments were performed using an open-source 3D printer with a custom-designed high-temperature printhead for extruding PCL. The use of a publicly available open source system makes our results easily replicable by other laboratories. Computational fluid dynamics (CFD) simulations of the extrusion process were also carried out to gain a more comprehensive understanding of the flow of the PCL inside the different nozzles used. We anticipate that our results will find practical applications in facilitating the configuration of AM systems for creating hybrid constructs in the shortest time without dismissing print accuracy.

## 2. Materials and Methods

### 2.1. Materials

Polycaprolactone (PCL, CAPA 6400; Perstorp, Sweden) with a mean molecular weight of 37 kDa was used as received from Perstorp Holding AB as the base polymer biomaterial for the scaffolds. The PCL has a melting point of 59 °C, and a melt flow index of 70.8–27 g/10 min^−1^ according to the provider’s reports. Poloxamer 407 (P407, Pluronic^®^ F127; Sigma-Aldrich, Madrid, Spain) was received from Sigma-Aldrich and used to prepare the hydrogels at 40 wt.%. P407 powder was added gradually to cold Milli-Q water to facilitate the dilution and stirred vigorously. Once the solution was homogenized, it was centrifuged and stored overnight at 4 °C to remove air bubbles. P407 was deposited interlaced with the PCL for the generation of hybrid scaffolds.

### 2.2. Open-Source High-Temperature Microextrusion-Based Printhead

The proposed printhead utilizes a 220 Vac band heater (25 × 25 mm^2^; LJXH, Shanghai, China) attached to a hollow aluminum block. A 5 mL stainless steel syringe (SSY-5E; Musashi Engineering, Tokyo, Japan) is inserted in an aluminum block (Figure S1). The syringe receives the pellets of PCL, and it is capable of working under elevated pressure and temperature. A 100 K thermistor (B57540G0104J; EPCOS AG, Malaga, Spain) is inserted into the aluminum block to measure its temperature. The thermistor is close to the syringe to obtain more accurate measurements (see Figure S1). The aluminum block insulation from the carcass is made employing cork sheets. Printing pressure is controlled using a 12 Vdc solenoid valve (EVT307-6D-02F-Q; SMC Inc., Tokyo, Japan) and a pressure regulator (AR20-N01BG-RYZ; SMC Inc., Tokyo, Japan). PCL extrusion was performed without a piston under compressed air pressure using a special syringe adapter (AT-5E-SUS; Musashi Engineering, Tokyo, Japan).

The printed parts of the printhead were designed using an open-source computer-aided design (CAD) software (FreeCAD; v0.17) [30] and exported as stereolithography (STL) files. The components were printed with a desktop 3D printer (Ultimaker 3; Ultimaker, Geldermalsen, Netherlands) in polycarbonate (Ultimaker PC; Ultimaker, Geldermalsen, the Netherlands). The STL files (Figure S2) of the printhead carcass and the fan support can be found as supplementary files (File S1) or downloaded from the NIH 3D Print Exchange repository [31]. Additional information about the final dimensions of the open-source printhead is available in Figure S3. The complete bill of materials is detailed in Table S1.

### 2.3. Measurement of the Temperature Response in the Extrusion System

Target operating temperatures of 100, 120, and 140 °C were established for the extrusion system, and several heating cycles were conducted to reach those temperatures. The temperatures selected represent the PCL temperatures of the experiments, which were set in the Repetier-Host software. The temperatures inside the aluminum block and the inner face of the printhead carcass were measured and monitored with two 100 K negative temperature coefficient (NTC) thermistors connected to the Rumba electronics. The temperature on the carcass (outside) was determined by using calibrated thermal images obtained with a thermal camera (Testo 871; Testo, Cabrils, Spain).

### 2.4. Open-Source 3D Printer Specifications and Main Configuration

The microextrusion-based printhead was installed into an open-source desktop 3D printer (Sigma R16; BCN3D Technologies, Barcelona, Spain), replacing the original FDM extruder. The printhead moves across the XY-axes (horizontal plane), while the build platform moved in the z-axis (vertical plane). According to the manufacturer’s specification, the mechanical resolution of the 3D printer is up to 12.5 μm. The original electronics of the printer were replaced by a Rumba board (Reprap Universal Mega Board with Allegro driver; RepRapDiscount, Hong Kong, China) connected to a 12 Vdc/30 A power supply. Both the printhead heater and the solenoid valve were controlled from the metal-oxide semiconductor field-effect transistor (MOSFET) terminals of the board. The 220 v heater used a relay (RLP/5-12D; MAHLE Electronics, Motilla del Palanca, Spain) connected to the 12 Vdc MOSFET controller (RepRapDiscount, Hong Kong, China). The custom modification of Marlin firmware (v1.1) was uploaded to control the whole printing system.

### 2.5. G-Code Generation and Printing Software

Three-dimensional (3D) printed PCL scaffolds were designed using open-source CAD software (FreeCAD; v0.17) and exported as STL files. The STL files generated were transformed into G-code through the open-source slicing software Slic3r (Slic3r; v1.2.9) and post-processed using custom Perl scripts. This pipeline allows for the modification of the original G-code to the particular requirements of our printhead. The modified G-code was uploaded to the 3D printer via Universal Serial Bus (USB) port using a free host software (Repetier-Host; v1.6.2), which was also employed to set the print temperatures and monitor the whole printing process.

### 2.6. 3D Scaffold Printing

The stainless-steel syringe was filled with pellets of PCL, and then heated to the target temperature for at least 15 min before starting the experiments. A fixed value of the pneumatic pressure of 700 kPa was applied to the PCL in all of the experiments. This value has proven to be sufficient for most of the PCL utilized in extrusion-based 3D printing [22,32].

**Figure 1:**
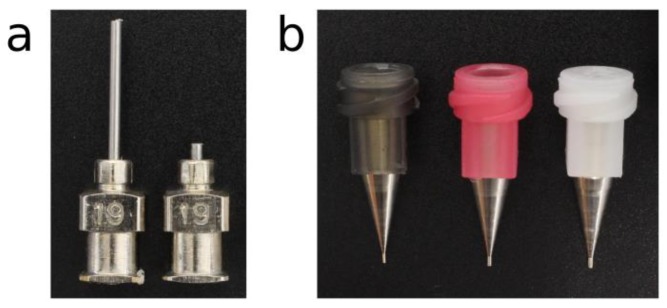
The nozzles selected for the experiments. (**a**) Stainless steel cylindrical standard (left) and shortened (right) nozzles of gauge 19 (G19). (**b**) Conical nozzles of gauge 30 (G30), 27 (G27), and 25 (G25) respectively, from left to right.

Conical nozzles (Micron-S dispensing tips; Fisnar Inc., Germantown, USA) and cylindrical nozzles (Stainless steel dispensing tips; Fisnar Inc., Germantown, USA) with different inner diameters (IDs) were utilized to print the PCL (Table 1). Shortened cylindrical nozzles with the same IDs as standard cylindrical nozzles were generated by cutting the needle from 13 to 2 mm (Figure 1).

### 2.7. Hybrid Construct Preparation

Hybrid constructs, as a proof-of-concept, were generated by depositing P407 and PCL strands together within the same construct layers. PCL strands were printed continuously into different zigzag patterns, while P407 strands were deposited independently next to the PCL strands. Conical nozzles with different IDs were selected for printing both biomaterials: 437 μm (Micron-S dispensing tips; Fisnar Inc., Germantown, USA) for PCL and 200 μm (Nordson EFD; Alfafar, Spain) for P407. The carriage (or printhead travel) speed was 14 mm s^−1^ for printing both PCL and P407 at print temperatures of 140 °C and 25 °C, respectively.

### 2.8. Printing Performance Metrics

**Figure 2:**
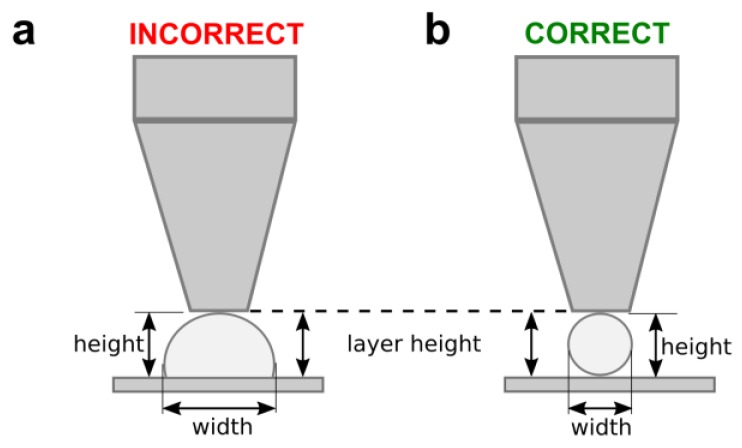
The scheme of the layer height adjustment used to evaluate the influence of the carriage speed on the PCL deposition. A slow speed produces wider and flattened filaments of PCL (**a**). The correct carriage speed should generate homogenous filaments of widths similar to that of the nozzle diameter (**b**).

Straight PCL filaments were printed onto a glass slide using different values of print temperature, nozzle diameter, and carriage speed. The same printing pressure (700 kPa) was set for all samples. In line with our previous works [33], and following the scheme of Figure 2, the layer height in the slicing software was set to the same value as that of the nozzle diameter. The strand roundness was calculated as the ratio between the measured height and width of the printed filaments. A circular section will have a roundness of 1.0, while irregular sections showing values lower than 0.3 will represent flattened strands.

PCL scaffolds were photographed right after being 3D-printed. The images of the scaffolds were processed with ImageJ software (NIH, Bethesda, MD, USA) [34] to measure the printed areas and the width and height of the strands. Porosity, in percentage, was calculated as the volume of a construct’s pores to its total volume [17]. The photographs and videos of the printing processes were taken using a digital single-lens reflex (DSLR) camera (EOS 700D; Canon Inc., Tokyo, Japan) placed on a firm tripod and under controlled lighting conditions. Images of PCL filament heights were taken using a USB microscope camera (KKmoon 500x; Digital microscope, Shenzhen, China).

We weighted the quantity of PCL extruded in 1 min *P* to calculate the volumetric flux of PCL. The volumetric flux is the rate of volume flow across a unit area as follows:

(1) Q=P/(60 ρ A),

where *A* is the outlet area and ρ is the density of PCL CAPA™ 6400 provided by the manufacturer (1100 kg/m^3^). *Q* was calculated to eliminate the influence of the different nozzle diameters and shapes on the results.

### 2.9. Computational Fluid Dynamics Model

A CFD simulation of the biomaterial flowing through different nozzle geometries was conducted in ANSYS^®^ Academic Research Fluent, Release 19.2 (ANSYS, Canonsburg, PA, USA). The extrusion of PCL was simulated as a non-isothermal steady-state process (discarding the melting process, the start-up, and the shutdown phases), coupling fluid-flow and heat-transfer problems. A two-dimensional (2D) axisymmetric model was chosen for the simulations, considering the volume-of-revolution characteristic of both the syringe and the nozzle. Different rectangular cell sizes were tested and the final mesh was selected for mesh independence of the solution, accuracy, and computational effort.

Conical, cylindrical, and shortened cylindrical nozzles with the same ID (437 μm) were simulated to evaluate the dynamics of PCL extrusion. The nozzles were modeled as stainless-steel parts that receive the PLC flow heated in the syringe. The heat transfer between the nozzle and the surrounding air was evaluated with an additional simulation using an extended domain. This simulation gave a mean heat transfer coefficient (HTC) between nozzle and air of 4 W/m^2^·K for an air-free stream temperature of 21 °C.

A pressure inlet boundary condition of 700 kPa was imposed at the upper part of the syringe. The nozzle output was set as a pressure outlet at atmospheric pressure, and a non-slip condition was imposed for all of the inner walls. The stainless-steel syringe, at a constant temperature of 120 °C, was considered as a thermally insulated component transferring heat to the incoming PCL fluid.

### 2.10. Constitutive Equations of the PCL

The pseudoplastic PCL CAPA 6400 was modeled as a generalized Newtonian fluid using the Bird–Carreau viscosity law [35,36]:

(2) $$\eta_{t}\left(\dot{\gamma }\right)=\eta_{0}{\left[1+{\left(\lambda \;\dot{\gamma }\right)}^{2}\right]}^{\frac{n-1}{2}},$$

where $\eta_{t}$ represents the viscosity law at some reference temperature $T_{ref}$, $\eta_{0}$ the zero-shear viscosity, $\dot{\gamma }$ the shear rate, λ the relaxation time, and *n* the degree of shear-thinning, which is a material-dependent factor. The parameters of the Bird–Carreau equation (Table 2) were extracted from the experimental data provided by the manufacturer, showing that this model fitted well the experimental data under those conditions (Figure S4).

The temperature dependence of the viscosity was modeled as:

(3) $$\eta =H\left(T\right)\;\eta_{t}\left(\dot{\gamma }\right),$$

where $\eta_{t}\left(\dot{\gamma }\right)$ is the viscosity law at some reference temperature $T_{ref}$, and H(T) is the Arrhenius law [37]:

(4) $$H\left(T\right)=exp\frac{E_{a}}{R}\;\left(\frac{1}{T-T_{0}}-\frac{1}{T_{ref}-T_{0}}\right)$$

where $E_{a}$ is the activation energy, *R* is the thermodynamic constant, $T_{0}$ corresponds to the lowest absolute temperature that is thermodynamically acceptable, and $T_{ref}$ is the reference temperature. $E_{a}$ (Table 2) was obtained from Equations (3) and (4) using a $T_{ref}$ of 100 °C and the zero-shear viscosity values provided by the manufacturer at 125 °C and 150 °C (152 Pa s and 95 Pa s, respectively).

## 3. Results and Discussion

### 3.1. Control of the PCL Temperature

**Figure 3:**
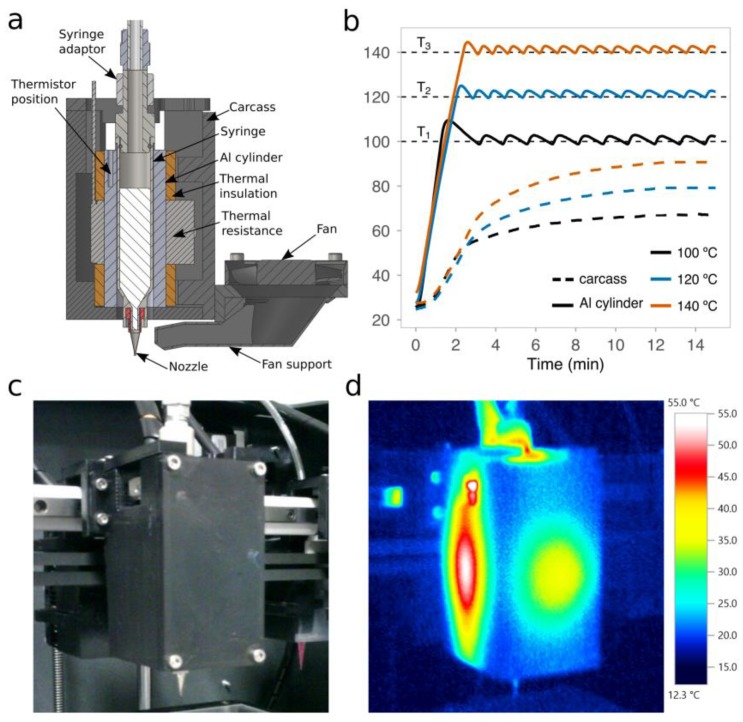
The open-source printhead for three-dimensional (3D) printing of polymers of a high melting point. (**a**) A schematic section view of the 3D computer-aided design (CAD) of the printhead with all of its components. (**b**) The transient response of the experimentally measured temperatures in the aluminum block and the interior of the carcass over time at three different target temperatures: 100 °C, 120 °C, and 140 °C. (**c**) Standard and (**d**) thermal images of the printhead installed in the 3D printer for a target temperature of 120 °C.

The deposition of PCL requires precise control of the print temperature to attain suitable viscosity values and optimal printing performance [25]. We first developed a new open-source microextrusion-based printhead that could provide this kind of control at short response times. The printhead was designed to smoothly fit in desktop 3D printers (Figure 3a) and control print temperatures ranging from 100 °C to 140 °C, far below the decomposition temperature of PCL (200 °C). Even if we established 140 °C as the upper limit of the printhead target temperatures, much higher temperatures can be reached with the same band heater and using different materials for the carcass, such as high-temperature photocrosslinkable resins or even Bakelite. After a series of experiments working under 100 °C (Figure 3b), temperature fluctuations in the aluminum block that surrounds the metallic syringe were only up to 2.5 °C. Therefore, thermal oscillations were very low over time and within the range of the expected levels.

The printhead carcass was made of PC, that is, a material that exhibits a heat deflection temperature of at least 110 °C. Therefore, we verified that the measured temperature on the inner face of the carcass was always below 110 °C (Figure 3b). Besides this, using a thermal camera, the temperature distribution across the external face of the carcass was also checked for a target temperature of 120 °C (Figure 3c,d). We found that the highest temperatures were in the middle of the faces with clear hotspots in the lateral ones. These results are consistent with our design, because, in order to reduce the printhead dimensions, the thermal insulation that resulted was thinner on the lateral faces than on the front and back ones (see final dimensions in Figure S3). However, a more compact design is beneficial, because it facilitates the installation of multiple printheads on the same carriage of the 3D printer.

### 3.2. Influence of Temperature, Shape, and Diameter of Nozzles on the PCL Flow Rate

**Figure 4:**
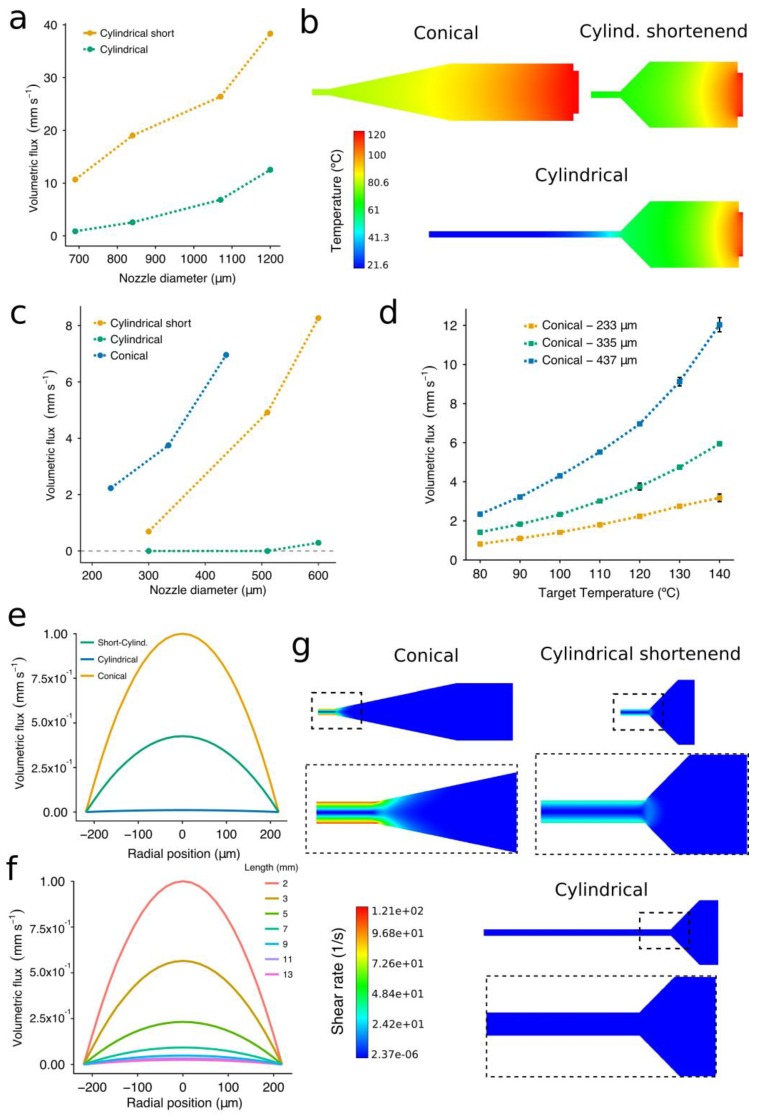
Experimental and simulated volumetric fluxes from the computational fluid dynamics (CFD) simulations using nozzles of different geometries and sizes. (**a**) The relationship between volumetric flux and needle length for cylindrical nozzles at a target temperature of 120 °C. (**b**) Temperature contour plots from the CFD simulation using conical, cylindrical, and shortened cylindrical (2-mm length) nozzles. (**c**) The relationship between volumetric flux and nozzle shape for IDs ranging from 690 μm to 1200 μm at a target temperature of 120 °C. (**d**) The relationship between volumetric flux and target temperature. (**e**) Velocity output profiles from the CFD model when using the three nozzle types with the same inner diameter. (**f**) Velocity output profiles from the CFD model of a G25 cylindrical nozzle with the needle length ranging from 13 mm to 2 mm. All the simulations were performed with a G25 conical nozzle at a target temperature of 120 °C, and an inlet pressure of 100 kPa. (**g**) Shear rate contour plots from the CFD simulation using conical, cylindrical, and shortened cylindrical (2-mm length) nozzles. The experimental data represent the mean and standard deviation of six experiments (n = 6).

Nozzle limitations regarding flow rate when extruding molten biomaterials were investigated using two nozzle geometries: cylindrical [21,32] and conical [24]. Flow rates are known to be higher under the latter nozzle type than under the former one [26,38]. However, we explored the potential benefits of using so-called shortened cylindrical nozzles, which have a needle of reduced length (Figure 1a). Shortened cylindrical nozzles exhibited significantly higher volumetric fluxes than the standard ones when extruding PCL at 700 kPa (Figure 4a). This may due to the decrease in the print temperature that comes along with the shorter needle length (Figure 4b). The difference was even greater when working with the large nozzle diameters. For instance, shortened cylindrical nozzles of inner diameter 690 μm and 840 μm generated a volumetric flux that was 7 and 12 times higher than the standard ones, respectively (Figure 4a). Besides this, no flow was observed when extruding PCL at a pressure of 100 kPa and a target temperature of 120 °C in the standard cylindrical nozzles with inner diameter lower than 600 μm (Figure 4c). Therefore, a heating cover should be installed in the tip of the dispensing syringe to avoid the dramatic temperature drop that increases the PCL viscosity. However, when building hybrid constructs, these high temperatures near the tip outlet might also affect the evaporation rate of the already-printed bioinks.

When printing PCL with nozzles of a small diameter (less than 510 μm), the conical nozzles always generated greater volumetric fluxes than the shortened ones (Figure 4c). Even if different gauges were employed, this trend held over the range of conical nozzle diameters included in this study. We also confirmed the significant variation in the flow depending on the diameter of the conical nozzle (Figure 4d). The volumetric flux increased more rapidly when printing with nozzles of a large diameter at high print temperatures (Figure 4d). For example, the volumetric flux of the G25 conical nozzle at 140 °C was around 5 times higher than that at 80 °C, while this difference was only around 4 times for nozzles of 335 μm and 233 μm. These results are meaningful because the higher the volumetric flux, the faster the carriage can move. Therefore, when printing with nozzles of a small diameter, even if we decided to increase the nozzle temperature in order to obtain a higher flow, the foreseen increase in volumetric flux would not be as significant as when using the nozzles with the largest diameter. Thus, we demonstrated that both nozzle shape and diameter were essential to facilitate the extrusion flow. Note that the values of the volumetric flux displayed above cannot be directly applied as print speeds for the 3D printing setup because these experiments were generated under constant pressure in free air, and not over a printing surface.

The flow rate of PCL not only depends on the nozzle geometry [39] but also on the evolution of the temperature along the nozzle. Overall, the results of the CFD simulations allowed us to foresee limitations in the extrusion process and gave us a more comprehensive understanding of the PCL flow inside the studied nozzles. The constitutive model incorporated the dependency of the PCL viscosity from the shear rate and the temperature using the Bird–Carreau viscosity law [36] along with the Arrhenius law [37]. A reasonable agreement was achieved between the experimental data and the simulations, with mean relative errors of 6% and 12% in the case of the temperature and the volumetric flux, respectively. The velocity profiles obtained at the tip of the three nozzles (Figure 4e) confirmed that the conical nozzles showed the highest volumetric flux for both large (Figure 4a) and small diameters (Figure 4d).

To assess the influence of the needle length on the extrusion rate, a cylindrical nozzle was simulated with the needle length ranging from 2 to 13 mm (Figure 4f). The standard cylindrical nozzles (a 13-mm length needle) are mostly exposed to the air, transferring their heat to the surroundings and producing a dramatic increase in the viscosity of the PCL. The velocity profiles showed the importance of these features in the extrusion of PCL, with a greater extrusion flow as the nozzle became progressively shorter. We would also like to point out that the registered velocity profiles (Figure 4e,f) follow the parabolic shape representative of Newtonian fluids, perhaps indicating a reduced influence of the shear rates on the PCL viscosity. This could be because the shear rate values during the extrusion were small in all cases (Figure 4g), so the PCL viscosity is always near the upper plateau, causing the Newtonian behavior. Similar to [38], the nozzle geometry with the highest shear rates was the conical one. In this case, all shear rate values were smaller than 121 s^−1^ and located in a region at 80 °C. In further investigation, we plan to check for possible inconsistencies in the model at low print temperatures (80–60 °C) where the plateau will be reduced because the departure point from the constant-viscosity regime will move towards the left.

We can conclude that the PCL volumetric flux at a constant target temperature and pressure is strongly dependent on the nozzle morphology that conditions the shear rate and print temperature at the nozzle tip. Consequently, the conical nozzles were revealed as the best choice concerning extrusion flow, while the shortened nozzles are preferred to the standard cylindrical ones unless the temperature along the nozzle is entirely controlled.

### 3.3. Determination of the Carriage Speeds for PCL Extrusion

It is well-known that not only do pore size and porosity have an impact on cell attachment and proliferation, but also scaffold architecture has a significant influence on tissue growth kinetics [40,41]. As the degradation of the scaffolds progresses, the size of their strands or filaments decreases while their pore size increases [6]. So, we need, above all, a precise control of the diameter of the scaffold strands just after printing. Therefore, we first examined the printed strands of PCL regarding section shape (roundness), dimensions, and discontinuities using conical nozzles, which exhibited the best performance in the previous section (Figure 4c).

**Figure 5:**
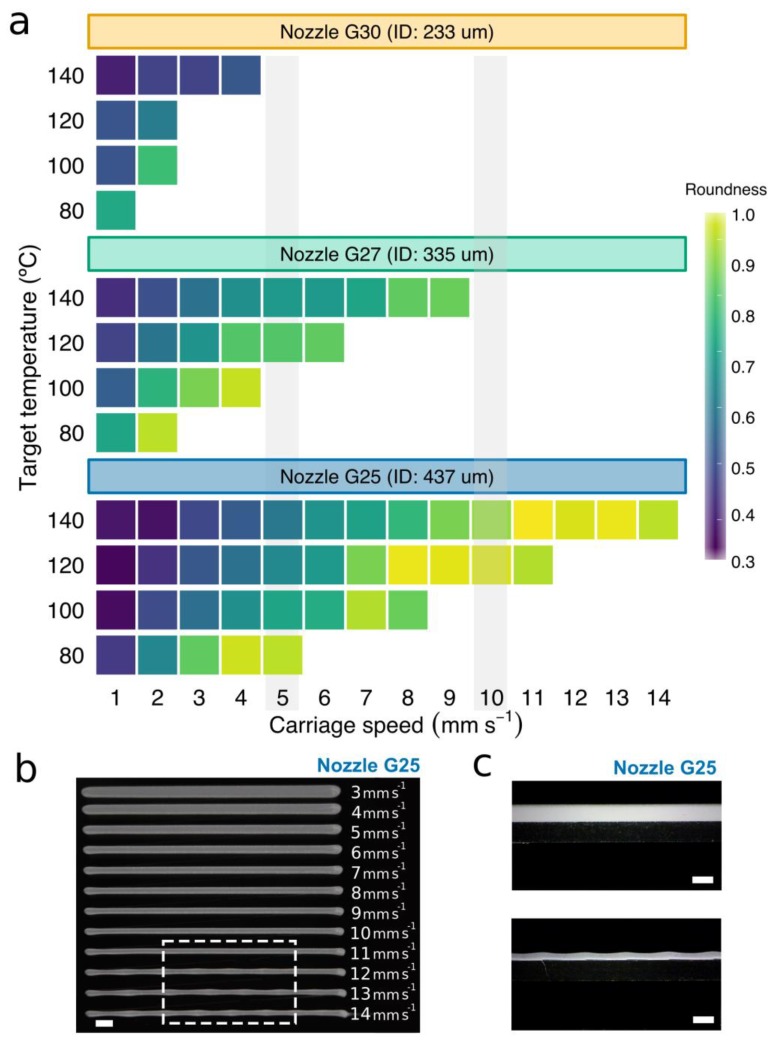
Evaluation of properties of the printed PCL strands. (**a**) Evolution of the roundness of the printed PCL strands versus the carriage speed for the three conical nozzles at different target temperatures. White areas are those in which strands of heterogeneous section are produced due to an excessive carriage speed. (**b**) A representative image of 12 filaments of PCL printed using a G25 conical nozzle at 120 °C and carriage speeds ranging from 3 to 14 mm s^−1^. The white dash box indicates the heterogeneous sections due to the excessive carriage speed. (**c**) Representative photographs of the height of PCL filaments printed using a G25 conical nozzle at 120 °C and carriage speeds of 15 mm s^−1^ (lower) and 1 mm s^−1^ (upper). Scale bars: 1 mm.

The higher the print temperature, the greater the carriage speed that could be used. The maximum carriage speed at 140 °C was 14, 9, and 4 mm s^−1^ for the G25, G27, and G30 conical nozzles, respectively. As the print temperature was lowered to 120 °C, carriage speeds have to be reduced by 21, 33, and 50% for the G25, G27, and G30 conical nozzles, respectively. The same trend was observed for 80 °C and 100 °C. Slow carriage speeds generated filaments of low roundness (Figure 5a), with the worst values (between 0.3 and 0.5) obtained at the lowest carriage speeds (1–2 mm s^−1^). This effect was even more pronounced when the target temperature was higher than 120 °C due to the low viscosity of the PCL. Visual examination of the printed filaments revealed excessive PCL deposition, with the material flattening against the print surface (Figure 5b,c). Similar results with a notable increase in the width of the PCL strands as the print temperature rose were reported by Sheshadri et al. [39].

The increase in roundness was directly proportional to the carriage speed, reaching values close to 1 in some cases. In addition, we observed that low target temperatures drastically reduced the range of potential values for the carriage speed. This could be mainly due to an increase in viscosity with a decrease in nozzle temperature, which was also evidenced by the constitutive equation (see Equation (3) and Figure S4).

**Figure 6:**
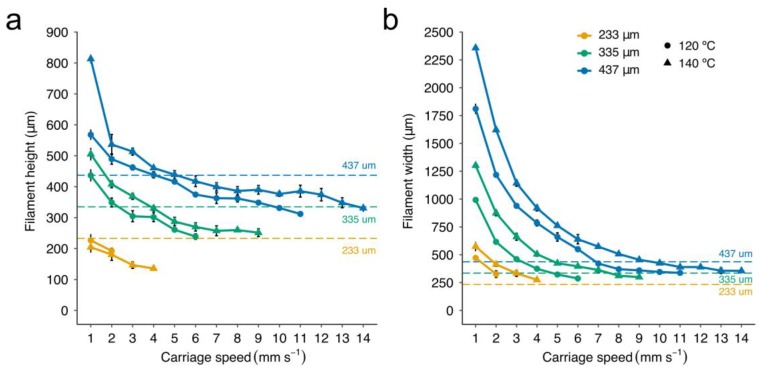
Evolution of the dimensions of the strands of PCL printed versus carriage speed. (**a**) The height and (**b**) the width of the strands for the three studied conical nozzles were measured at a pressure of 700 kPa and target temperatures 120 °C and 140 °C. Data represent the mean and standard deviation of six different samples (n = 6).

When carriage speeds are higher than a particular threshold, small fluctuations in this speed may easily lead to inconsistencies in the extruded material (see the waves at the bottom of Figure 5b). In these cases, the PCL was deposited either discontinuously or in strands of heterogeneous section (Figure 5c). Note that the threshold for the smallest conical nozzle working at low temperatures was so low (around 2 mm s^−1^) that there was very little room for maneuver. On the other hand, PCL filaments were not only more rounded at high carriage speeds, but also almost always smaller than the nozzle ID (Figure 6a,b). This effect was more visible when lowering the target temperature from 140 to 120 °C. These results agreed with the observations presented by Shim et al. [23], where strand widths ranging from 275 μm to 90 μm were generated using a nozzle of ID 200 μm. We conclude that homogeneous strands of small diameter can be printed with nozzles of large gauge (>G20) at a much faster speed (Figure 6a). This situation is beneficial for our aim of creating hybrid constructs because the higher the speed, the lower the print time results.

### 3.4. Evaluation of PCL Print Times

Time is one the most critical problems in biofabrication because it affects productivity, cell viability, and limits the construction of large-scaled tissues, which will ultimately be mandatory if real tissue replacements want to be generated [42]. When bioprinting hybrid constructs, there is a notable increase in the time required due to their inherent complexity. Long print times, including the preparation of the bioinks, will severely decrease cell viability, burdening the future of the tissue construct because the cell nutrients and oxygen requirements might not be reached on time [29].

**Figure 7:**
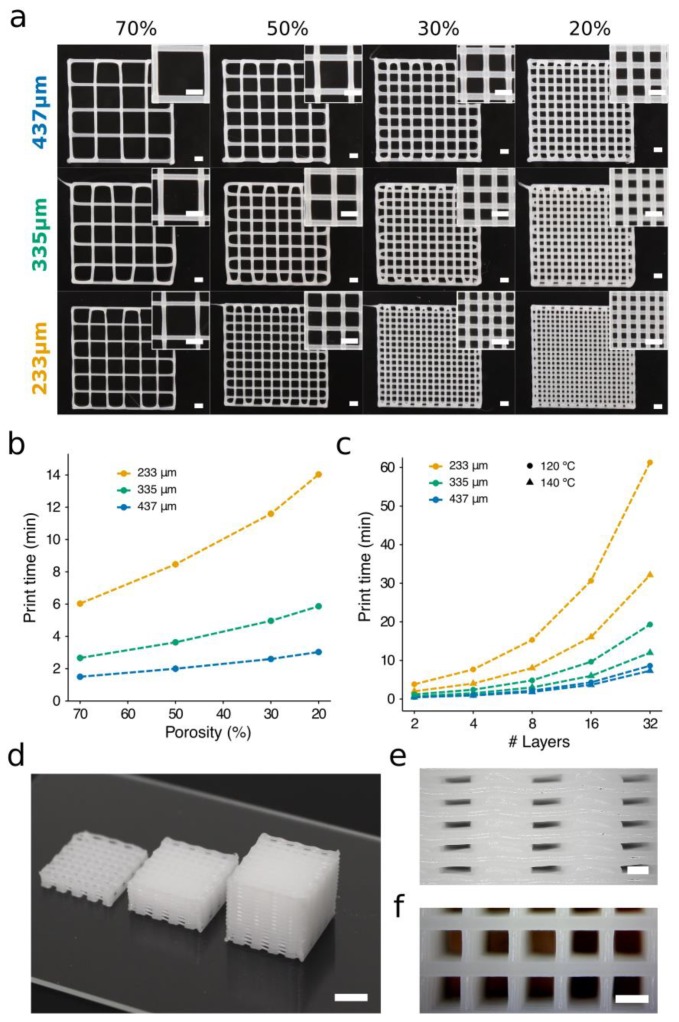
The influence of porosity percentage and layers stacked on the print time. (**a**) Two-layered PCL porous scaffolds of porosity ranging from 70 to 20% were printed using conical nozzles of three diameters at a target temperature of 120 °C and a pressure of 700 kPa. The travel speed of the printhead was adjusted to make the width of the strands match the nozzle ID (233 μm: 2 mm s^−1^; 335 μm: 5 mm s^−1^; 437 μm: 7 mm s^−1^). Scale bars: 1 mm. (**b**) Print times to produce a 12 x 12 mm scaffold of eight layers and different degrees of porosity at 120 °C. The carriage speeds selected to produce strands of 335 μm diameter were 2, 5, and 11 mm s^−1^ for 233-, 335-, and 437-μm nozzles, respectively. (**c**) Print times for a 12 mm square side scaffold, with 30% porosity and several layers stacked. Maximum print speeds were utilized for each nozzle–temperature configuration. (**d**) PCL porous structures created by stacking 8 (left), 16 (center), and 32 (right) layers. Scale bar: 5 mm. Side (**e**) and top (**f**) views of 3D scaffold of 32 layers printed with a 437-μm nozzle at 140 °C and a speed of 14 mm s^−1^. Scale bars: 500 μm.

To study these matters, scaffolds of different porosity were generated using conical nozzles of three distinct diameters (Figure 7, Video S1). The target temperature was kept constant (120 °C), and we adjusted the carriage speeds to each nozzle. The carriage speeds were selected according to the results displayed in Figure 5a and Figure 6 with the widths of the strands matching nozzle IDs. These values were 2, 5, and 7 mm s^−1^ for the 233-, 335-, and 437-μm nozzles, respectively. Note that smaller nozzles require more trajectories per layer than the large ones to generate scaffolds of similar porosity, as the latter create thicker strands than the former. A higher number of trajectories also implies longer print times, which is particularly important when creating hybrid scaffolds. For instance, creating the scaffold of porosity 20% with the nozzle of ID 437 μm required 2.6 min, while the 335-μm and 233-μm nozzles required 5.9 min and 19.6 min, respectively. Our results confirmed the linear relationship between porosity and print time (Figure 7b). The implications of these factors should be analyzed carefully when looking for significant reductions in the print time of PCL scaffolds.

**Figure 8:**
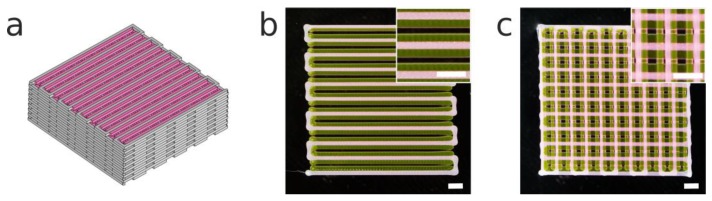
(**a**) A CAD model of a 3D hybrid construct of dimensions 20 × 20 mm. (**b**) The first layer and (**c**) stack of two layers printed of the hybrid construct. It is composed of PCL (ID: 437 μm; 14 mm s^−1^; 140 °C; 700 kPa) and intercalated filaments of poloxamer P407 (ID: 200 μm; 14 mm s^−1^; room temperature (RT); 100 kPa). Scale bars: 2 mm.

As shown in the previous section, it is possible to generate scaffolds with the same strand diameter using different nozzle sizes. For instance, printing the scaffolds in the middle row of Figure 7 (ID 335 μm) can be done in three different ways. In Figure 7, the most obvious solution for creating strands of diameter 335 μm is to employ a combination 335 μm/5 mm s^−1^ (ID/carriage speed). It would also be feasible to produce these strands with 233 μm/2 mm s^−1^ or even with 437 μm/11 mm s^−1^. The three options would provide the same diameter (Figure 8a), but the largest nozzle (437 μm) will demand the shortest print times.

During PCL 3D printing, it is necessary that each layer solidifies (or at least gains enough consistency) before the next layer is stacked. Otherwise, as more layers are stacked, the whole scaffold will deform as a consequence of standing on their own weight in a non-consistent state. This aspect will depend on such features as PCL properties, layer dimensions, processing temperature, print speed, and strand diameter. Within these experiments, the PCL solidification times varied depending on the utilized printing temperature, with 120 °C and 140 °C requiring longer times. When using these printing temperatures with slow print speeds (from 1 to 4 mm s^−1^), no additional cooling was required for an accurate layer stacking. However, when print speeds were higher than 4 mm s^−1^, a layer fan was utilized to enhance the cooling of deposited PCL strands (Figure S5). The layer fan was attached to the printhead using a 3D-printed fan support that directed the air flow to the printed construct. The enhanced cooling enabled the use of faster print speeds without scaffold deformations when multiple layers were stacked (Figure 7d–f).

The production of scaffolds with a clinically relevant size requires a compromise between print speed and resolution. We studied the scalability by printing the same porous scaffold (Video S2), i.e., the same number of trajectories, but increasing the number of stacked layers. Figure 7c illustrates the influence of the carriage speed over the total print time per scaffold. The carriage speed selected for each nozzle was the highest possible at 120 °C (233 μm: 2 mm s^−1^; 335 μm: 6 mm s^−1^; 437 μm: 11 mm s^−1^) and 140 °C (233 μm: 4 mm s^−1^; 335 μm: 9 mm s^−1^; 437 μm: 14 mm s^−1^). We observed that the highest speeds, which are associated with large nozzles, permitted faster scaffold generation. For example, the production of a porous scaffold with 32 layers stacked and printed at 120 °C would require 10 additional minutes if using a 335-μm nozzle instead of a 437-μm nozzle (Figure 7c). However, if the 233-μm nozzle is utilized instead, the print time increases to 52 min. The great time differences obtained revealed that using non-optimized printing parameters would introduce inadmissible lead times into the bioprinting process, which would ultimately result in a great decrease in cell viability when hybrid scaffolds are produced. It is important to note that when using nozzles of a small diameter and at a medium-low temperature (120 °C), the print times increased by more than 100% (Figure 7c). In contrast, the time differences using nozzles of a large diameter at low or high temperatures can be neglected. Consequently, nozzles of a large diameter and low print temperatures are preferred to obtain short print times and assure high cell viability in hybrid constructs.

### 3.5. Proof of Concept of a Hybrid Construct using the Selected Print Parameters

One of the main advantages of using hybrid constructs that integrate soft hydrogels and rigid scaffolds is to provide better mechanical properties and a biological microenvironment suitable for cell survival and growth [43]. A 3D hybrid construct employing PCL and P407 was proposed as a proof of concept (Figure 8a). We used a multimaterial bioprinting system previously presented by the authors [33] with the new printhead incorporated into the same system. The calibration method proposed in the mentioned publication was critical for the alignment of both biomaterials (PCL and P407). We adjusted the print parameters according to the previous results and we were able to print both materials at the same carriage speed (14 mm s^−1^). Note that if a cell-laden bioink were to be employed, temperatures lower than 140 °C could be used with the G25 conical nozzle without substantially increasing the print time (Figure 7c).

In Figure 8b, we observe that the first layer of the hybrid construct showed a successful alignment of both materials, with homogeneous strands one beside the other, while Figure 8c presents the high shape fidelity of the hybrid construct. The use of PCL provided a stiffer framework for the incorporation of the P407 than if only a soft hydrogel had been used.

## 4. Conclusions

Herein, a novel open-source pneumatic printhead for PCL deposition was developed. We demonstrated its good capabilities in terms of temperature response and printing accuracy. This device helps us make our results easily replicable by other laboratories. Then, we presented a series of experiments that provide information useful to finding the best setup for PCL deposition in terms of time efficiency and print accuracy. Nozzle shapes were first analyzed through flow experiments and CFD simulations. The results demonstrate that the internal nozzle morphology represents one of the key points to be considered in the extrusion of PCL. Conical nozzles were revealed to be the best shape to achieve high print speeds, with significant flow differences for the different cylindrical shapes.

Assuming that the carriage speed must be always maximized without reducing the quality of the printed constructs, we explored the optimal values for the parameters nozzle size and print temperature. Print temperature is a limiting factor, as temperatures that are too high drastically increase the number of dead cells when depositing the next layer of the scaffold onto the already-printed layer of cells and bioink. On the other hand, temperatures that are too low result in difficulties in obtaining a proper PCL flow.

The scalability was studied by printing PCL scaffolds of different porosities and numbers of layers. This allowed us to detect the print parameters that have a direct influence on the print time. The results revealed that varying the nozzle diameter and target temperature led to time reductions of up to 50 min. Our results are important in TE because reducing the print times of hybrid constructs is crucial for building scaffolds of a clinically relevant size at high cell viability.

## Figures and Tables

**Table 1:** The conical and cylindrical nozzles utilized in the experiments with their corresponding gauge and inner diameter (ID).

Type	Inner Diameter (ID) μm and Gauge									
Conical	233	-	335	437	-	-	-	-	-	-
Cylindrical	-	300	-	-	510	600	690	840	1070	1200
	G30	G24	G27	G25	G21	G20	G19	G18	G17	G16

**Table 2:** The Bird–Carreau parameters and activation energy value used in the viscosity model of PCL at a $T_{ref}$ of 100 °C.

Bird–Carreau Parameters			Activation Energy (*E_a_*) kJ mol^−1^
Zero-Shear Viscosity (*η*_0_) Pa s	n	*λ*	
291.3	0.8	0.0083	30.7

## Supplementary Materials

The following are available online at http://www.mdpi.com/1996-1944/12/4/613/s1. Figure S1. Dimensions of the aluminum block located inside the polycarbonate carcass of the printhead for heating the 5 mL stainless steel syringe (see scheme on the right side). Figure S2. Images of the 3D CAD models (STL files) of the 3D printed printhead components. (a) Printhead_carcass.STL; (b) Front_cover.STL; (c) Syringe_cover.STL; (d) Fan_support.STL. Figure S3. Main dimensions of the open-source high-temperature printhead. Figure S4. The viscosity versus shear rate relationship of CAPA 6400. Points represent the experimental data provided by the manufacturer for temperatures 100 °C and 125 °C. Solid lines are the Bird–Carreau model fittings calculated according to Equation (3). Figure S5. Images of the fan support installed on the printhead to increase the cooling rate of the PCL. Table S1. Bill of materials of the printhead, including quantity, cost, description, and provider of each component. Video S1: Porous construct printed in PCL with 10 layers stacked using a 437-μm nozzle and an 11 mm s^-1^ print speed. Video S2: Porous constructs printed in PCL and P407 with different nozzles and carriage speeds. PCL: 233 μm, 4 mm s^−1^ (black); 335 μm, 9 mm s^−1^ (pink); and 437 μm, 14 mm s^−1^ (white). P407: 200 μm, 14 mm s^−1^ (transparent). File S1. 3D-printed STL files of the open-source printhead.
