# Supplementary material for: Efficient Fabrication of Polycaprolactone Scaffolds for Printing Hybrid Tissue-Engineered Constructs
Source: Materials (Basel). 2019 Feb 18;12(4):613. doi: 10.3390/ma12040613 (PMC6416605; doi:10.3390/ma12040613)
Supplement: Supplementary file 1 [file materials-12-00613-s001.pdf]

materials

Supplementary

Efficient Fabrication of Polycaprolactone Scaffolds for Printing Hybrid Tissue-Engineered Constructs

Enrique Sodupe-Ortega [1], Andres Sanz-Garcia [2,3,*], Alpha Pernia-Espinoza [1], and Carmen Escobedo-Lucea [2,*]

1 EDMANS Group, Department of Mechanical Engineering, University of La Rioja, San José de Calasanz 31, Edificio Departamental, 26004 Logroño, Spain; enrique.sodupeo@unirioja.es (E.S.-O.); alpha.pernia@unirioja.es (A.P.-E.)
2 Division of Pharmaceutical Biosciences. University of Helsinki. Viikinkaari 5 E (P.O. Box 56), 00014 Helsinki, Finland
3 Department of Mechanical Engineering, University of Salamanca, ETSII, Avda, Fernando Ballesteros, 2, 37700 Béjar (Salamanca), Spain
* Correspondence: andres.sanz-garcia@helsinki.fi (A.S.-G.); carmen.escobedo-lucea@helsinki.fi (C.E.-L.); Tel.: +358-294159326 (A.S.-G.); +358-294159329 (C.E.-L.)

Received: 30 December 2018; Accepted: 11 February 2019; Published: date

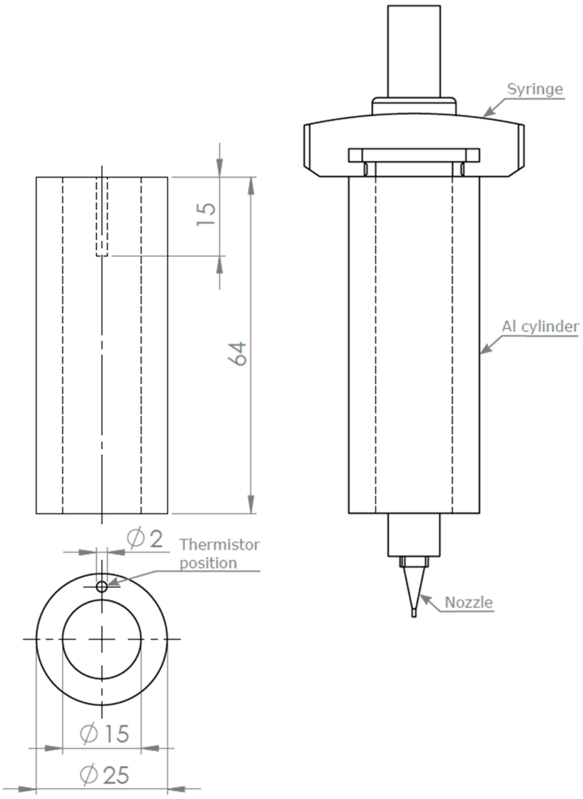

Figure S1. Dimensions of the aluminum block located inside the polycarbonate carcass of the printhead for heating the 5 mL stainless steel syringe (see scheme on the right side).

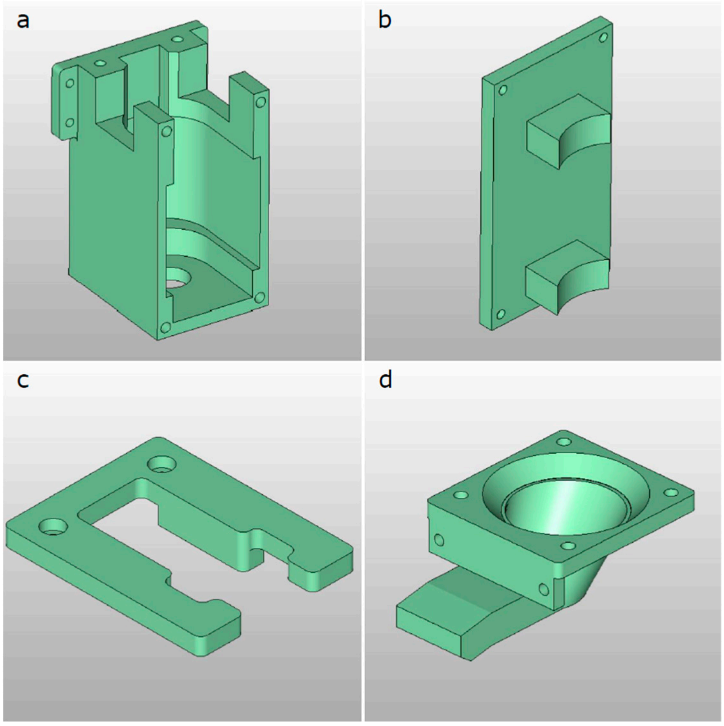

Figure S2. Images of the 3D CAD models (STL files) of the 3D printed printhead components. (**a**) Printhead_carcass.STL; (**b**) Front_cover.STL; (**c**) Syringe_cover.STL; (**d**) Fan_support.STL.

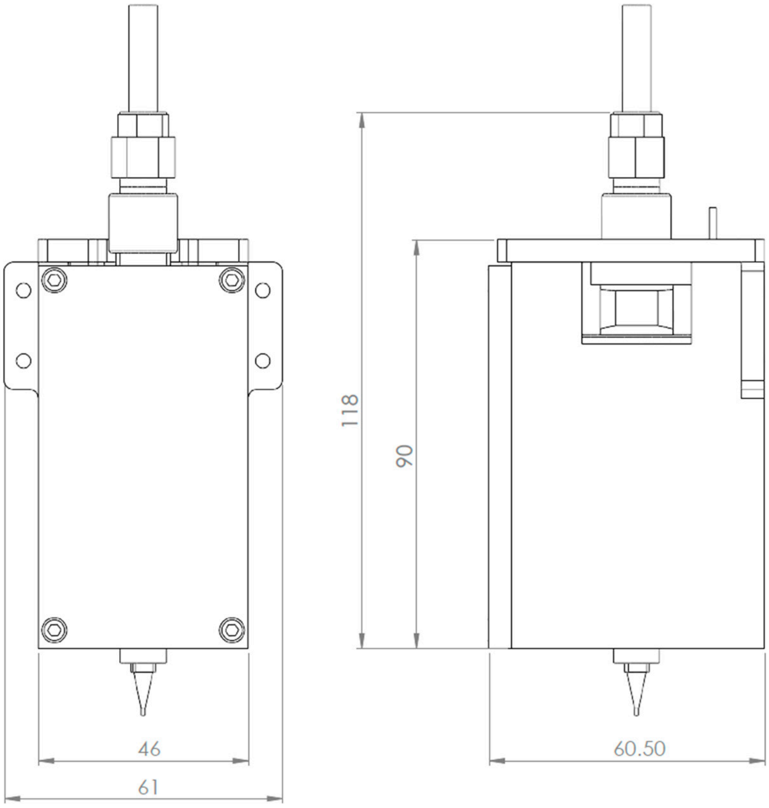

Figure S3. Main dimensions of the open-source high-temperature printhead.

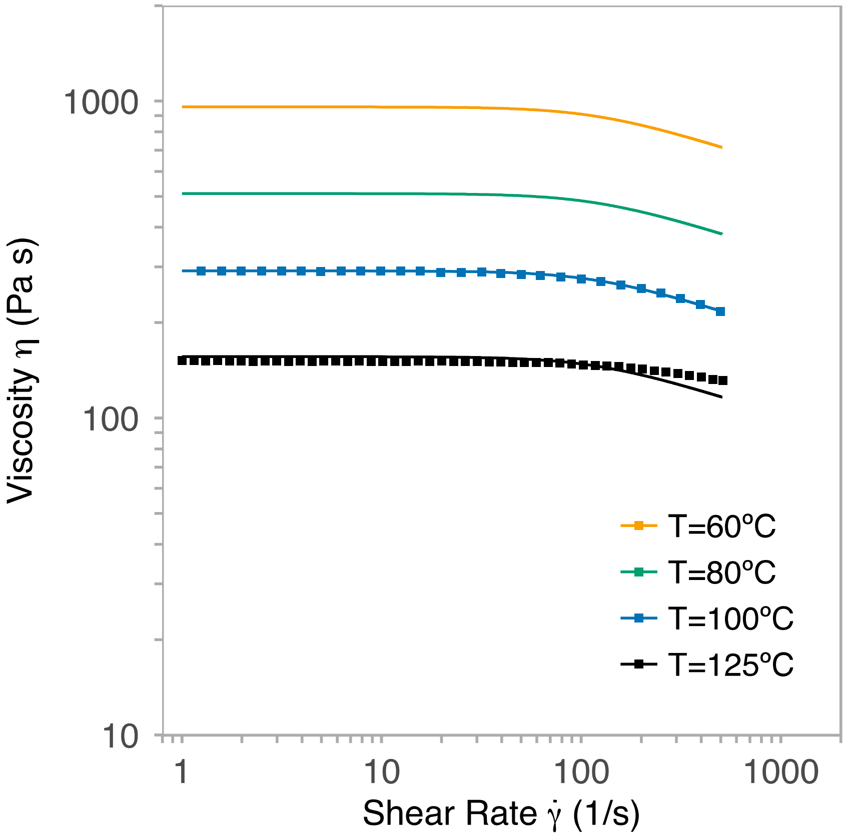

Figure S4. The viscosity versus shear rate relationship of CAPA 6400. Points represent the experimental data provided by the manufacturer for temperatures 100 °C and 125 °C. Solid lines are the Bird-Carreau model fittings calculated according to Equation (3).

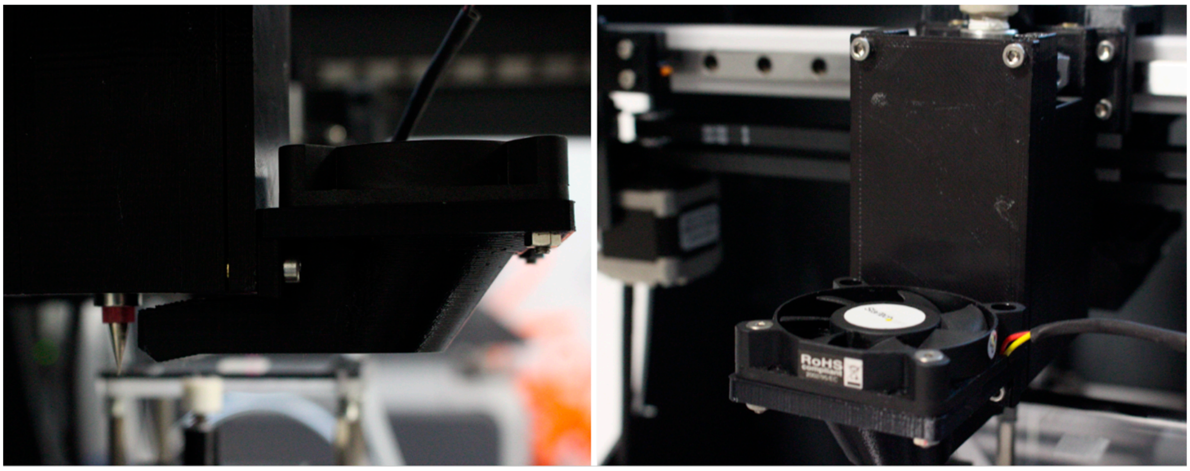

Figure S5. Images of the fan support installed on the printhead to increase the cooling rate of the PCL.

materials MDPI

Table 1. Bill of materials of the printhead, including quantity, cost, description, and provider of each component.

Part Name	Quantity	Cost (US$)	Description	Provider	Code
Printhead carcass	1	1.03	3D printed	3D printer	-
Front cover	1	0.20	3D printed	3D printer	-
Syringe cover	1	0.35	3D printed	3D printer	-
Aluminum block	1	13.49	SMC[a]	Misumi	-
M3 brass insert	6	1.52	SMC[a]	RS Online	278-584
Band heater 220V (25 × 25mm)	1	7.00	SMC[a]	LJXH	32856328124
Relay 12V-40A	1	3.00	SMC[a]	Nagares	RLP/5-12D
Thermistor NTC 100k	1	1.56	SMC[a]	RS Online	528-8592
M3x10 screw	6	2.86	SMC[a]	RS Online	660-4636
M3x25 screw	4	1.92	SMC[a]	RS Online	304-4435
Solenoid valve	1	51.95	SMC[a]	RS Online	EVT307-6D-02F-Q
Pressure regulator	1	19.70	SMC[a]	RS Online	AR20-N01BG-RYZ-A
Total		84.88			

[a] SMC = Standard Mechanical Component

Video S1: Porous construct printed in PCL with 10 layers stacked, using a 437 µm nozzle and an 11 mm s^{-1} print speed.

Video S2: Porous constructs printed in PCL and P407 with different nozzles and carriage speeds. PCL: 233 µm, 4 mm s^{-1} (black); 335 µm, 9 mm s^{-1} (pink); and 437 µm, 14 mm s^{-1} (white). P407: 200 µm, 14 mm s^{-1} (transparent).

File S1. 3D printed STLs files of the open-source printhead.
